# Gene expression profiles of mouse spermatogenesis during recovery from irradiation

**DOI:** 10.1186/1477-7827-7-130

**Published:** 2009-11-19

**Authors:** Fozia J Shah, Masami Tanaka, John E Nielsen, Teruaki Iwamoto, Shinichi Kobayashi, Niels E Skakkebæk, Henrik Leffers, Kristian Almstrup

**Affiliations:** 1 University Department of Growth and Reproduction GR-5064, Rigshospitalet, Blegdamsvej 9, DK-2100 Copenhagen O, Denmark; 2 Institute for Animal Experimentation, St. Marianna University Graduate School of Medicine, 2-16-1 sugao, Miyamae-ku, Kawasaki 216-8511, Japan; 3 Department of Pharmacology, St. Marianna University School of Medicine, 2-16-1 sugao, Miyamae-ku, Kawasaki 216-8511, Japan; 4 Center for infertility and IVF, International University of Health and Welfare Hospital, 537-3 Iguchi, Nasushiobara 329-2763, Japan

## Abstract

**Background:**

Irradiation or chemotherapy that suspend normal spermatogenesis is commonly used to treat various cancers. Fortunately, spermatogenesis in many cases can be restored after such treatments but knowledge is limited about the re-initiation process. Earlier studies have described the cellular changes that happen during recovery from irradiation by means of histology. We have earlier generated gene expression profiles during induction of spermatogenesis in mouse postnatal developing testes and found a correlation between profiles and the expressing cell types. The aim of the present work was to utilize the link between expression profile and cell types to follow the cellular changes that occur during post-irradiation recovery of spermatogenesis in order to describe recovery by means of gene expression.

**Methods:**

Adult mouse testes were subjected to irradiation with 1 Gy or a fractionated radiation of two times 1 Gy. Testes were sampled every third or fourth day to follow the recovery of spermatogenesis and gene expression profiles generated by means of differential display RT-PCR. In situ hybridization was in addition performed to verify cell-type specific gene expression patterns.

**Results:**

Irradiation of mice testis created a gap in spermatogenesis, which was initiated by loss of A1 to B-spermatogonia and lasted for approximately 10 days. Irradiation with 2 times 1 Gy showed a more pronounced effect on germ cell elimination than with 1 Gy, but spermatogenesis was in both cases completely reconstituted 42 days after irradiation. Comparison of expression profiles indicated that the cellular reconstitution appeared equivalent to what is observed during induction of normal spermatogenesis.

**Conclusion:**

The data indicates that recovery of spermatogenesis can be monitored by means of gene expression, which could aid in designing radiation treatment regimes for cancer patients leading to better restoration of spermatogenesis.

## Background

Treatment of cancers often includes radiation and/or chemotherapy and in many cases leads to temporally discontinuation of spermatogenesis. In particular treatment of testicular tumors leads to impaired spermatogenesis. Fortunately, fertility and preservation of androgen production can be sustained in many cases due to reconstitution of the seminiferous epithelia. Side effects of chemotherapy and radiotherapy however include cardiovascular disease, secondary malignancy and a reduced fertility [1]. Current knowledge about re-initiation of spermatogenesis after radiation is however limited, but could benefit the patient's chance of regaining fertility and proper androgen production.

Spermatogenesis is a long, complex and finely tuned process [2]; during this process, the developing germ cells are sensitive to endogenous and exogenous stress. Cancer therapies such as radiation and chemotherapy can cause temporary or permanent impairment of fertility in male cancer patients who usually are in the reproductive age [3-5]. Therefore, an important goal of successful treatment is to minimize the cytotoxic impact of the treatment in order to maximize chances of re-initiating spermatogenesis while still efficiently killing cancerous cells. To this end, it is necessary to understand how radiation affects the differentiating germ cell and thus produce infertility in male mammals.

Spermatogenesis is initiated from the most primitive type of spermatogonia, the type A-single (A_s_) or stem cell spermatogonium, which has two possible fates: self-renewal or committed differentiation [6]. The A_s _spermatogonia give rise to A-pair (A_pr_) and then A-aligned (A_al_) spermatogonia which are then able to differentiate into A_1_, A_2_, A_3_, A_4_, intermediate (In), and B spermatogonia [7]. When a type B spermatogonia enter the last mitotic division, it generates two primary spermatocytes, which initiate meiosis by replicating the DNA before they pass through a number of stages, that ends with the two nuclear divisions distinguished as meiosis I and II [8]. After the meiotic divisions each primary spermatocyte results in the formation of four haploid round spermatids [9]. The spermatids proceed through a long differentiation process (designated spermiogenesis) resulting in the release of spermatozoa.

Several studies have investigated the effect of irradiation on the testis. As early as in the 1950s Oakberg demonstrated in mice that type In and type B spermatogonia were very sensitive to irradiation while undifferentiated type A spermatogonia had a variable sensitivity [10,11]. More recent studies further demonstrated that A_1 _through A_4_, which are undergoing differentiation and are actively proliferating are the most radio-sensitive spermatogonia followed by A_pr _and A_al _spermatogonia. A_s _are the most radio-resistant spermatogonia, but they nevertheless show moderate sensitivity to radiation and alkylating agents [12-15]. In accordance, Dym and Clermont [16] found in rat that a fraction of type A spermatogonia, which gives rise to recuperation of the germ cell population, is particularly resistant to irradiation [17]. Spermatogonia are highly susceptible to DNA damaging agents, which block their mitotic activity and kill cells during the S-phase [3,14,15]. Since DNA damage leads to apoptosis when they try to divide, spermatogonia are more vulnerable than quiescent Sertoli and Leydig cells and spermatids, however spermatocytes that are in the meiotic divisions are also less vulnerable than spermatogonia [18,19].

Virtually the entire population of spermatogonia will die if exposed to sufficiently high X-ray doses and especially a fractionated irradiation [6]. Recovery may however be increased at very high doses with a fractionated irradiation [20]. After exposure to irradiation, spermatocytes and spermatids continue normal development and ultimately leave the testis as spermatozoa. If stem cells (A_s _spermatogonia) survive the irradiation, they may in some cases quickly initiate the recovery of spermatogenesis and repopulate the seminiferous epithelium [13,21]. The remaining A_s _spermatogonia will either first replenish their own numbers before they enter spermatogenic differentiation and in time, spermatogenesis spreads along the length of the tubule [22-24], or they can remain "arrested" in the testis as isolated spermatogonia in atrophic tubules [25,26]. In some cases a delay before spermatogenesis reinitiates has been observed [27,28].

Currently there is little evidence for damage to the somatic elements of the testis after moderate doses of radiation or chemotherapy. However, as the germ cells are dependent on Sertoli cells for survival, it is difficult to assess whether it is germ cells or somatic cells that are damaged by radiation. A recent study in rat testes demonstrated that radiation-induced block in spermatogonial differentiation may in fact be caused by damage to the somatic environment, i.e. the Sertoli cells, and not to the germ cells [29]. Indeed transplantation of Sertoli cells into irradiated testes has shown to stimulate recovery of endogenous host spermatogenesis [30]. Stimulation might however be indirectly as the endocrine androgen-estrogen balance seems crucial in stimulating spermatogonial recovery [31].

In the present study we aimed at implementing the tight link between gene expression profiles during the first postnatal wave (induction) of spermatogenesis and cell types present in the testis, to describe changes in the cellularity during recovery from irradiation. We generated expression profiles of several genes during testicular recovery from irradiation and deduced the cellular expression by *in situ *hybridization, which allowed us to follow the gap created in spermatogenesis. We define the gap size and compare the effect of 1 Gray (Gy) and 2 × 1 Gy on cellular changes and show that recovery effectively can be followed by means of gene expression.

## Methods

### Mice testis preparation

Male C3H/He strain mice were obtained from Japan SLC (Shizuoka, Japan). All animals were maintained under controlled conditions (22 ± 2°C, 55 ± 5% humidity, 12 h light/dark cycle, lights on 0600 h) and were given laboratory chow (CE-2, Japan Crea, Tokyo, Japan) and water ad libitum.

Eleven-week old mice were anesthetized with pentobarbital and covered with lead sheeting except a part of the scrotum. The testes were locally exposed to X-ray radiation with 1 Gy or 2 times 1 Gy with an interval of 7 days. Testes from 1 or 4 mice were sampled every third or fourth day during recovery where the testes were removed and weighted. One testis was fixed in 4% paraformaldehyde in 0.1 M phosphate buffer, pH 7.4, overnight at 4°C and subsequently dehydrated in graded series of ethanol and embedded in paraffin for *In situ *hybridization (ISH). The contralateral testis was snap-frozen in liquid nitrogen and used for preparation of total RNA. Mice testes radiated with 1 Gy were sampled on days 3, 7, 10, 14, 17, 21, 24, 28, 31, 35, 38, 42, 45, 48, 52, 56, 59, and 63 post-irradiation (pi). Testes irradiated to 2 × 1 Gy were first sampled after the second dose (7 days after first dose) and on days 7, 10, 14, 17, 21, 24, 28, 31, 35, 38, 42, 45, 48, 52, 59, and 63 pi. The dosing of irradiation was chosen based on literature to make sure that cellular change occurred.

Animal studies were approved by The Japanese Pharmacological Society and the animals were treated according to generally accepted guidelines for animal experimentation at St. Marianna University Graduate School of Medicine and guiding principles for the care and use of laboratory animals.

### Differential display analysis

Differential display (DD) was performed essentially as previously described [32]. Total RNA was purified from testes using the NucleoSpin RNA II kit as described by manufacturer (Macherey-Nagel, Düren, Germany). cDNA was synthesized using one-base-anchored AAGCTTTTTTTTTTTC (AAGCT_11_C) downstream primers. The cDNA was used in competitive polymerase chain reactions (PCRs) using two-base-anchored primers (AAGCT_11_CC) in combination with two different upstream primers. The *Tnp2 *band was displayed by the upstream primer CATAGAAATGGCGGACA; and *Vps26a*, *Gata1 *and *Ribc2 *were all displayed by the same upstream primer (ATCCTTGTGCCTCAGTT). *Dazl *were displayed with GATCATCTCTGCTA in combination with HT11G. The PCR products were separated on polyacrylamide gels, and quantified by phosphorimaging as described earlier [32]. Bands of interest were excised from the gel, re-amplified using the same upstream primer as in the competitive PCR and a HT_11_CC/HT_11_G primer with an additional T7-promoter overhang. This facilitated subsequent sequencing and identification of the excised band [32]. Quantification as measured by disintegrations per square mm was normalized to the background and thus expressed as intensity in arbitrary units.

### *In situ *hybridization

ISH was carried out as previously described [33]. ISH probes were designed from the DD DNA fragments and prepared by re-amplification of the fragments using nested primers specific to the mRNA, extended by a T3-promoter sequence, in combination with a downstream primer extended with T7-promoter sequence. The resulting PCR product was purified on a 2% low-melting agarose gel and sequenced from both ends using primers complementary to the added T3- and T7-promoter sequences. Aliquots of 200 ng were used for *in vitro *transcription with incorporation of biotin-labeled nucleotides using the MEGAscript-T3 (sense) or MEGAscript-T7 (antisense) kits as describes by the manufacturer (Ambion, Houston, TX). Tissue sections (8 um) were deparaffinized, re-fixed in 4% paraformadehyde (PFA), treated with proteinase K (P-2308, Sigma, St. Louis MO USA) (1.0-5.0 μg/ml), post fixed in PFA, pre-hybridized 1 h at 50°C, and hybridized o. n. at 50°C with biotinylated antisense and - sense control probes. Excess probe were removed with 0.1 × SSC (60°C) 3 × 33 min. Visualization was performed with streptavidin conjugated with alkaline phosphatase (1:1000) (Cat. No. 1093266, Roche Diagnostics GmbH, Mannheim, Germany) followed by a development with BCIP/NBT, for details se [33].

## Results

Two groups of 63 mice were irradiated with either 1 Gy or 2 × 1 Gy with an interval of one week. Recovery of spermatogenesis was followed in a period of about 60 days post irradiation (pi) by analysis of differentially expressed transcripts using Differential Display (DD) with primer combinations that previously had been applied to study postnatal testicular development [34]. ISH was in addition performed to verify the cellularity of the differentially expressed transcripts, since reduced expression at the whole-testes-level in most cases reflect the absence of the cells normally expressing the gene [34].

### Testicular weight after irradiation

**Figure 1:**
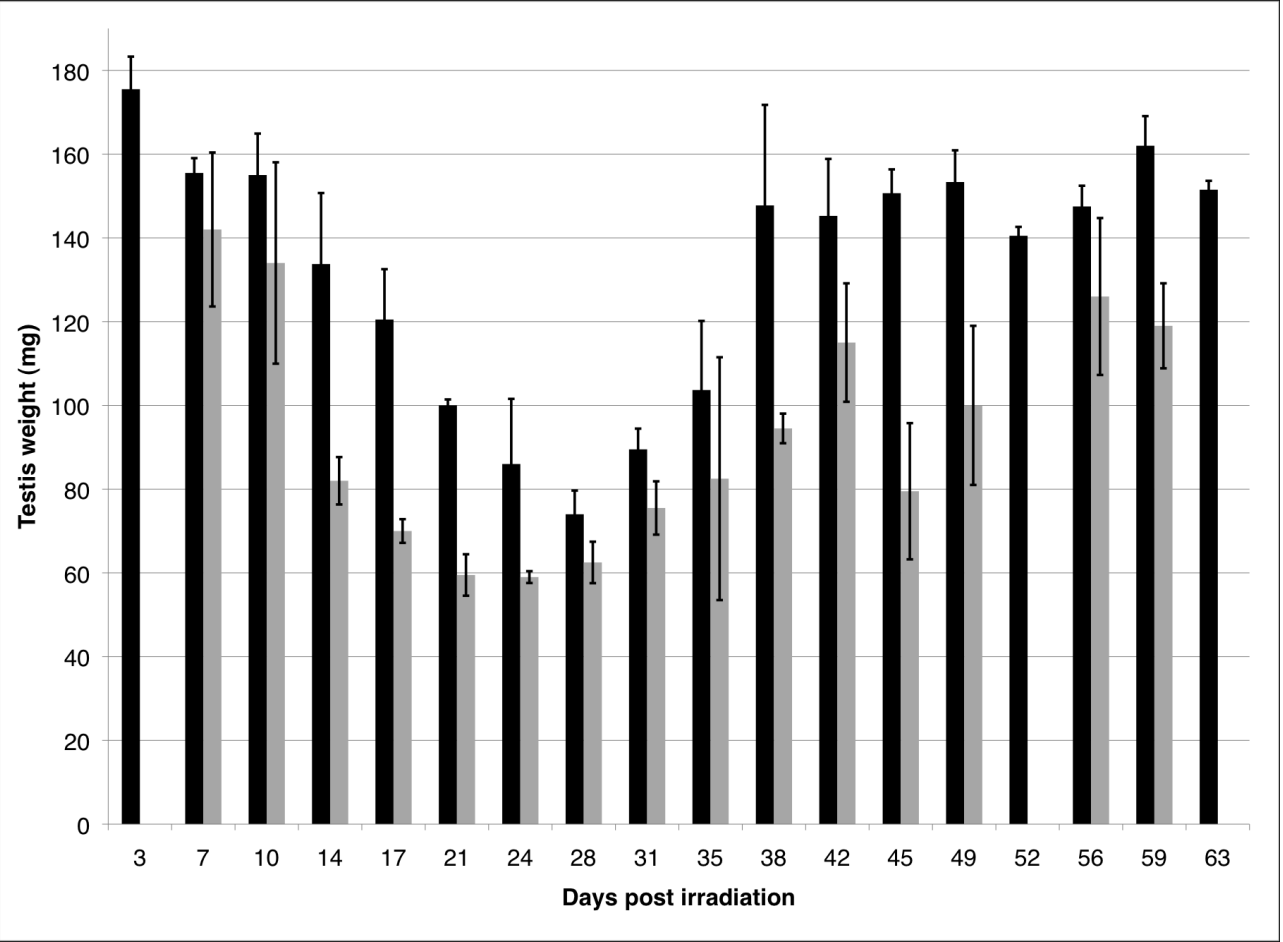
**Correlation between testis weight (mg) and time (days) after irradiation**. The testes of mice were locally exposed to radiation with 1 Gy (black columns) or 2 × 1 Gy with an interval of 7 days (light grey columns). The testes were weighted and sampled on the indicated days. For 2 × 1 Gy the days correspond to days after the second dose. Error bars represent standard deviation of the mean of the testes (n = 2-8) removed at the indicated day.

Irradiation with 1 Gy caused a gradual decrease in weight (fig. 1, black columns) reflecting elimination of germ cell populations. At post-irradiation (pi) day 3, the testis weight was in average 175 mg but gradually decreased to 75 mg until pi day 28. From pi day 28 the weight gradually increased and on pi day 63 the testis weight had returned to a level similar to its pre-treatment level (approximately 175 mg).

Approximately the same pattern was observed for mice testis exposed to 2 × 1 Gy (fig. 1, light grey columns). On pi day 7 the weight of testis was around 140 mg, which declined until it reached 60 mg on pi day 24, from where it gradually increased to a level similar to its initial weight. Thus, both treatments resulted in a significant decrease in testis weight and as expected, 2 × 1 Gy had a stronger effect on testis weight than a single dose of 1 Gy.

The profile of testicular weight during recovery is presented as it essentially follows the profile obtained from genes expressed in pachytene spermatocytes (see below).

### Changes in gene expression after irradiation

We have previously identified a large number of transcripts that were specific to distinct germ cell types [34]. The transcripts were originally identified by DD analysis of gene expression during normal pn development (the first wave of spermatogenesis). ISH was subsequently used to precisely identify the expressing cell types, which lead to the identification of three distinct clusters of gene expression profiles. The first cluster of up-regulation corresponded to the appearance of pachytene spermatocytes and the second to round spermatids, while genes in the down regulated cluster were expressed in Sertoli cells [34]. Based on this dataset, we selected primer combinations that would show changes in expression levels of mRNAs from each of the three clusters in order to follow the elimination and recovery of these distinct cell types after irradiation. We analyzed the effects of irradiation on a range of genes, but since all genes that belong to the same cluster show a similar profile, we only show one gene from each cluster. In addition, we also examined one gene expressed in spermatogonia.

The gene 'deleted in azoospermia-like' (*Dazl*; MGI:1342328) was chosen to represent genes expressed in spermatogonia/spermatocytes, vacuolar protein sorting 26 A (*Vps26a*; MGI:1353654) to represent genes expressed in pachytene spermatocytes (first cluster), transition protein 2 (*Tnp2*; MGI:98785), representing genes expressed in haploid round and elongating spermatids (second cluster) and GATA binding protein 1 (*Gata1*; MGI:95661) as an example of genes expressed in Sertoli cells.

### Expression of the spermatogonia/spermatocyte-specific *Dazl *after irradiation

**Figure 2:**
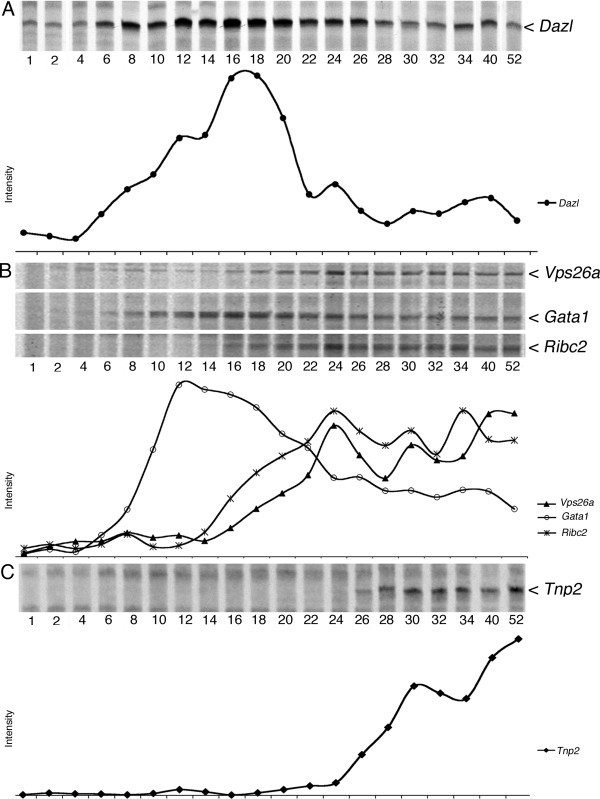
**Gene-expression data during postnatal development**. Fragments from DDRT-PCR reactions are displayed on long polyacrylamide gels and transcripts identified by sequencing. Intensity of bands was quantified by phosphor imaging. **A**) Autoradiogram displaying a band corresponding to *Dazl*, a spermatogonia and spermatocyte-specific gene and below quantification of the band. **B**) Autoradiogram and quantification of bands corresponding to *Vps26a*, *Gata1 *and *Ribc2 *all generated by the same combination of primers and thus displayed on the same gel. *Vps26a *and *Ribc2 *are both examples of genes expressed in pachytene spermatocytes, while *Gata1 *is expressed in Sertoli cells. **C**) Autoradiogram and quantification of the spermatid-specific gene, *Tnp2*.

Initially we confirmed the *Dazl *expression profile in untreated testis during pn development (fig 2A). *Dazl *was lowly expressed until pn day 6 wherefrom it increased gradually until pn day 18. After pn day 18 the expression decreased rapidly and reached a stable level on pn day 26 where it remained in adult mice. This profile thus represents a typical gene expressed in spermatogonia and early spermatocytes.

**Figure 3:**
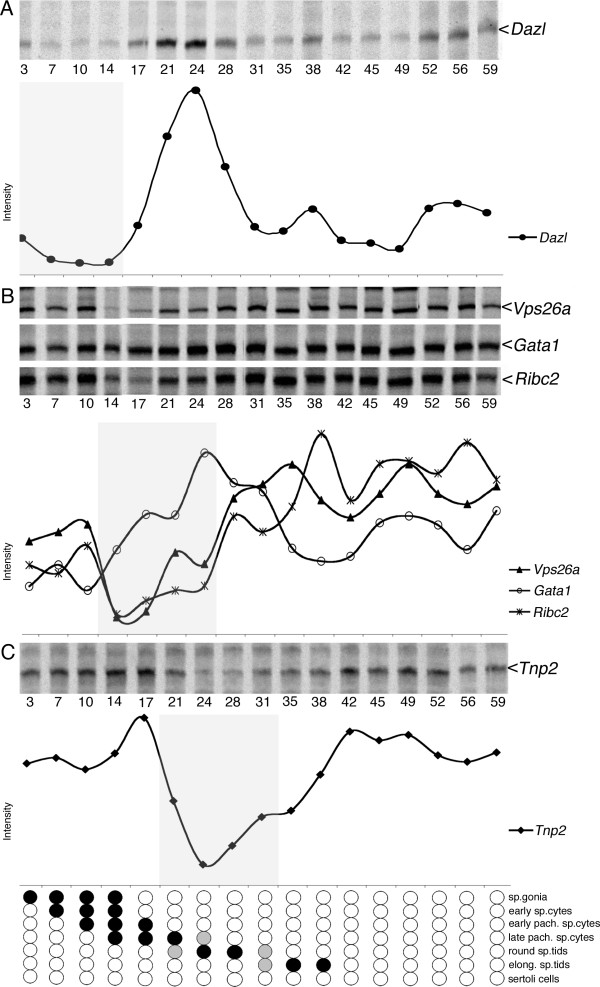
**Expression profiles after irradiation with 1 Gy**. Autoradiograms and quantifications after irradiation with 1 Gy. The same genes as is figure 2 are displayed: **A**) *Dazl*, **B**) *Vps26a*, *Gata1*, *Ribc2*, and **C**) *Tnp2*. Grey areas indicate roughly the absence of the cell types that express the gene, except for *Gata1*. Below an estimation of the cell types affected by irradiation as spermatogenesis recovers. Open circles indicate that the indicated cell type is present, while filled circles indicate that the indicated cell types are absent. Grey circles indicate that the cell type is partial present.

**Figure 4:**
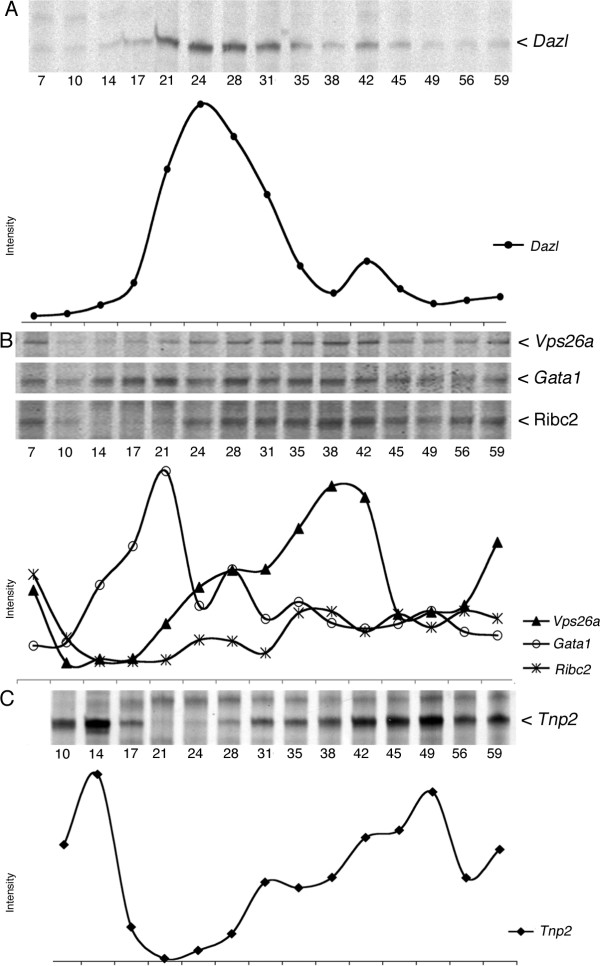
**Expression profiles during recovery after fractionated irradiation**. Autoradiograms and quantifications after fractionated irradiation with two times 1 Gy. The same genes as is figure 2 and 3 are displayed: **A**) *Dazl*, **B**) *Vps26a*, *Gata1*, *Ribc2*, **C**) *Tnp2*.

Next, we investigated the expression of *Dazl *during recovery after exposure to irradiation with two different doses (fig 3A &4A). Expression of *Dazl *after irradiation with 1 Gy resulted in a profile showing a small decreased from pi day 3 to 14 followed by a steep increase at pi day 17 peaking at pi day 24 and again followed by a gradual decrease to reach the initial level at pi day 31. Approximately, the same profile was observed with a fractionated radiation (fig. 4A) even though values reaching the initial level first were acquired at pi day 35.

### Expression of the pachytene spermatocyte-specific *Vps26a *after irradiation

As for *Dazl*, we first confirmed that the *Vps26a *expression profile in untreated testis corresponded to expression in a specific germ cell type, in this case pachytene spermatocytes (fig. 2B). The expression of *Vps26a *was initially very low until pn day 16 from whereon it increased gradually to reach its maximum level on pn day 24 where it remained (with smaller oscillations) in adult mice. Thus *Vps26a *represents a typical late cluster 1 gene expressed in pachytene spermatocytes; which was also verified by ISH (Additional file 1).

During recovery from irradiation with 1 Gy, the expression of the *Vps26a *transcript was initially very high, but on pi day 14, its expression decreased dramatically and it remained low until pi day 17; this was followed by a recovery which reached the initial level at pi days 28-31 (fig. 3B).

Irradiation with 2 × 1 Gy had a stronger effect on the expression of *Vps26a*, where the level decreased dramatically from pi day 7 to 10 and remained low on pi days 10-17. From pi day 17 the level increased gradually to reach a level (at pi day 38) that was considerably higher than the initial level (day 7). However, this was followed by a slight decline and on pi day 45 the level had returned to the initial level (fig. 4B). Approximately, the same expressions profile was observed for another cluster 1 gene, *Ribc2 *(fig. 4B and table 1) even though quantification levels were different for the two. One exception was however pi day 59, which probably was quantified too high for *Vsp26a *as the autoradiogram, did not seem to reflect the high quantification.

**Table 1:** A survey of the analyzed genes

	*Dazl*	*Ribc2*	AK029908* (1700028K03Rik)	*Vps26a*	Spata3*	*Tnp2*	*Gata1*
Mouse Genome Database ID	1342328	1914997	1923671	1353654	1917310	98785	95661
Transcript expressed in (according to ISH)	B-spermatogonia- preleptotene Spermatocytes Stage VI - VII	Pachytene Spermatocytes Stage VI - VII	Pachytene Spermatocytes Stage VI - VII	Late- to diplotene Spermatocytes Stage IX - XI	Round Spermatids Stage VII - VIII	Round Spermatids Stage VII - VIII	Sertoli cells
Gene classified as [34]	NA	Standard Cluster 1	Standard Cluster 1	Late Cluster 1	Cluster 2	Cluster 2	Sertoli
Day of appearance during induction of spermatogenesis	6 - 8 pn	16 pn	14 pn	16 - 18 pn	28 pn	28 pn	8 pn
Day of re-appearance after irradiation to 1 Gy	17 pi	28 pi	28 pi	28 pi	38 pi	38 pi	17 pi

* Data not shown

### Expression of the spermatid-specific *Tnp2 *after irradiation

During pn development *Tnp2 *exhibited an expression profile that was compatible with expression in round and elongating spermatids. *Tnp2 *was until pn day 26 not expressed, but then at pn day 28 highly up-regulated to a level where it remained in adult mice (fig. 2C). This corresponds to expression in round spermatids. ISH in addition verified that *Tnp2 *only was detectable in round spermatids from stage VII (step 7) to elongating spermatids in stage VIII to stage XI. However, in stage XI (step 11) *Tnp2 *was down-regulated and from stage XII and in elongated spermatids the transcript was not detectable (Additional file 2). This showed that *Tnp2 *corresponded to a cluster 2 gene, but with a relatively restricted expression.

Expression of *Tnp2 *in testis irradiated with 1 Gy showed a gradual increase and reached its maximum on pi day 17 (fig. 3C). This was followed by a dramatic decrease and on pi day 24 the expression level was reduced to the lowest level. This was followed by a gradual recovery and the expression returned to the initial level (pi day 3) at pi day 42 (fig. 3C).

A similar pattern was observed for *Tnp2 *after exposure to 2 × 1 Gy (fig. 4C). Initially the expression increased with the highest level on pi day 14, from where it decreased dramatically to an almost undetectable level that persisted until pi days 21-24. From pi day 28 the level gradually increased and reached the highest level on pi day 49. Even though some oscillation was observed in the quantified level, this suggest that the recovery after irradiation was prolonged for testis exposed to 2 × 1 Gy as compared to a single dose of 1 Gy. This may however be caused by the experimental setup where counting of pi days was started 7 days after the first irradiation in the 2 × 1 Gy group (see discussion).

**Figure 5:**
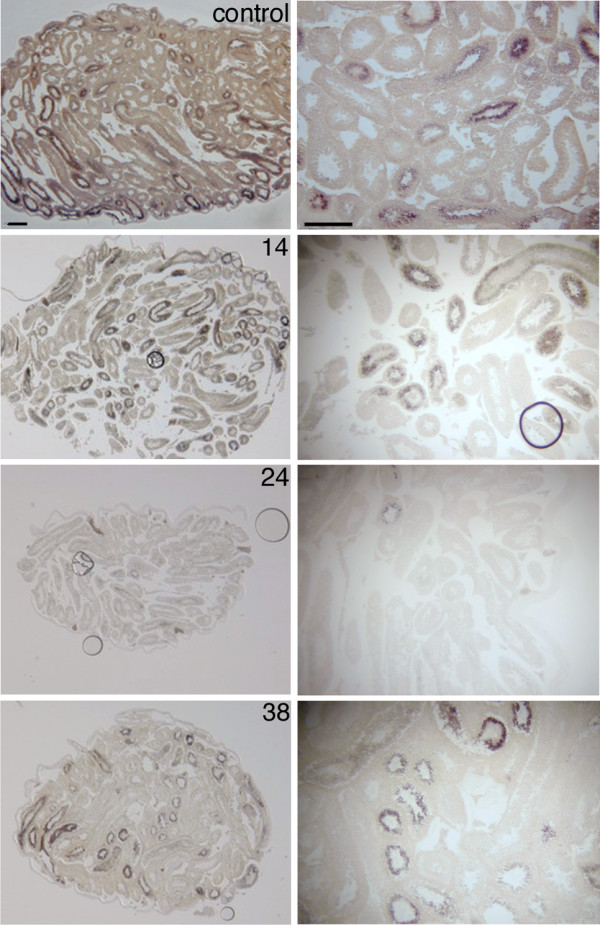
**in situ hybridization analysis of *Tnp2 *expression during recovery after irradiation**. ISH analysis of *Tnp2 *expression during recovery in adult mouse testis after irradiation with 1 Gy. A control, pi days 14, 24 and 38 were shown. Low magnification images of whole testis are shown (left) together with a higher magnification of a representative part of the testis (right). The bars correspond to 100 μm. ISH from additional pi days can be found in Additional file 3.

To confirm the observed *Tnp2 *expression profile, we performed ISH on adult 1 Gy irradiated mouse testis using an ISH probe corresponding to the DD fragment. In the untreated adult mouse testis (control) we observed about 50% of tubules expressing *Tnp2*. Until pi day 14, the morphology and the number of *Tnp2*-expressing tubules were similar in control and irradiated testis (fig. 5). However, from pi day 17 the number of tubuli with spermatids expressing *Tnp2 *was markedly reduced and on pi day 24 the *Tnp2 *transcript was essentially absent (fig. 5 & Additional file 3). At this time the morphology of the testis was clearly affected by the irradiation. *Tnp2 *expressing tubules re-appeared on pi day 31 even though the morphology of the testis was still abnormal (Additional file 3). The number of expressing tubules increased gradually and on pi day 49 approximately 60% of tubules expressed the *Tnp2*. On pi day 56 and 63 spermatogenesis apparently had completely recovered since the number of tubules expressing *Tnp2 *was similar to that in untreated adult testis (Additional file 3).

### Expression of Sertoli cell-specific *Gata1 *after irradiation

The expression of *Gata1 *confirmed to Sertoli cells was determined both during normal pn development and after irradiation (figs. 2B, 3B &4B).

During normal development the expression profile showed that *Gata1 *expression initially was very low, but from pn day 6 the level was dramatically up-regulated to reach a maximum on pn day 12-14, which was followed by a gradual decrease and from pn day 28 it remained constant at a relatively low level.

Next, we examined the expression of *Gata1 *during recovery after irradiation with a dose of 1 Gy. *Gata1 *was initially expressed at a relatively low level until pi day 14 from where it increased and remained high until pi days 24-31, followed by a decline until pi day 35 where its expression level fluctuated around the initial level (fig. 3B).

Finally, we investigated the effect of 2 × 1 Gy on *Gata1 *(fig. 4B). As for the 1 Gy experiment, expression of *Gata1 *increased gradually after radiation and on pi day 21 it was at its maximum. From pi day 24 the expression rapidly decreased and on pi day 59 the expressions level had decreased to the initial level (pi day 7) (fig. 4B).

The results are summarized in table 1.

## Discussion

### The effect of irradiation on testes weight

To evaluate the potency of different levels of radiation we first investigated the correlation between testis weight and the time of recovery. We found that after irradiation with 1 Gy, the weight was reduced to less than half of the initial (unaffected) weight at pi day 28 (fig 1, black columns), whereas for testis exposed to 2 × 1 Gy the weight had apparently decreased to less than half already at pi day 21 (fig 1, light grey). Since pi day 0 for the mice exposed to 2 × 1 Gy was set to be the day of the second treatment (7 days after the first irradiation), the observed shift in the timing of pi weight decrease fits the difference receiving the first dose of radiation and the timing was thus probably the same for the two treatments. Results are in line with what have been reported earlier with the same doses [24]. Even though no statistical test was performed, the results however indicted that an extended period of reduced testis weight and a slightly larger weight loss is observed for irradiation with two consecutive irradiations and thus probably more complete removal of the radiosensitive cells. This is expected since the two successive irradiations imply loss of two consecutive populations of radiosensitive spermatogonia. In both cases spermatogonial stem cells were however able to re-populate the seminiferous tubules with apparently normal germ cells. The time-window with the largest weight-loss (fig. 1 dark gray bars; pi day 14 to 28 for 1 Gy) corresponds essentially to the period where the large pachytene spermatocytes are absent (fig. 3B shaded area; pi day 14 to 24).

### Cells killed by irradiation

The loss of testis weight and subsequent regain reflect a period of continuous loss of cells followed by reconstitution of the testis to a level reflecting normal weight. In an earlier study we used gene expression to describe the cellular changes that occur during induction of normal spermatogenesis during pn development (the first wave of spermatogenesis) [34]. The vast majority of differential expression, at the whole testis level, during induction of spermatogenesis could be attributed to changes in the cellularity of the testis. Thus, up-regulation of a gene was tightly linked to the appearance of a specific cell type and down-regulation linked to dilution of exiting cell types [34]. In this study we took advantage of the close correlation between gene expression and germ cell types to describe the changes in cellularity that occur after irradiation. Others have used similar doses in earlier studies [23,24] and their histological description in general fits with what we observed by ISH analysis (see below).

Expression profiles of the germ cell-specific genes *Tnp2 *and *Vps26a *were used to determine which subpopulation of the spermatogonial stem cells were killed by irradiation. As the timing of each stage of the seminiferous epithelium is known [2] it is possible to calculate which cells were affected by the irradiation prerequisite that the re-population observed after irradiation is similar to what is observed during induction of spermatogenesis during pn development. Thus, the number of days from the irradiation (pi day 0) to the observed decline in expression was used to "count backward" from the affected cells (equaling drop in cell type-specific expression) to reach an estimation of which cells originally must have been killed/affected by the irradiation.

*Tnp2 *was found to be down-regulated around pi day 21 with 1 Gy (fig. 3C) and it is known that this gene is highly expressed in spermatids in stages VII-XI [35,36](Additional file 2). Subtraction of 21 days from stage VII step 7 suggest that the affected cells should be B spermatogonia in stage V. From the *Vps26a *expression profile we know that it is highly expressed in late spermatocytes and early spermatids corresponding to stages IX-III (fig 2B and Additional file 1) and after irradiation with 1 Gy the expression drops off at pi day 14 (fig 3B). Subtracting 14 days from stage IX again suggest that the radiation-sensitive cells were B spermatogonia in stage IV - V. However, due to the sampling intervals of 3-4 days we cannot exclude that In and A1-A4 spermatogonia also were affected. Data from 2 × 1 Gy leads to similar conclusions and thus there seems not to be any differences in the initially affected cell-types.

We conclude that the cells affected by irradiation were A1 through B spermatogonia, which is in line with earlier results [11-15]. This implies that the spermatogonial stem cells (A_s_) repopulate the seminiferous tubules after radiation in a similar fashion to induction of spermatogenesis during normal pn development.

We can however not tell anything about the nature of the effect on the spermatogonia. Recent reports have indicated that the effect of irradiation is on the Sertoli cells and not the germ cells leading to failure of nursing the germ cells [29,30]. Likewise alterations in the androgen-estrogen balance can significantly boost the recovery of spermatogenesis [31] indicating that the endocrine balance, which Sertoli cells are part of, is of crucial significance. Very recently gene expression patterns in irradiated LBNF rats (devoid of germ cells) treated with different combinations of hormone modulating drugs was investigated. This identified affected genes in the somatic compartment of the recovering testes that thus possible was involved in modulating the hormonal influence of recovery [37]. The study was however in a mutant germ-cell-less mouse model and combination of the present results and similar study designs could identify if the same set of genes is found in recovering testes from normal mice. Yet other mechanisms (i.e. chromosomal damage and repair) may influence the recovery after irradiation.

### The length of the spermatogenesis "gap" created by irradiation

Based on the expression profile of the examined genes we were able to estimate the length of the gap introduced by irradiation. The gap is defined as the time-differences between when a gene disappears and reappears (from down- to up-regulation) and implicit the time-window from when specific cell types are eliminated and subsequently reappears. An observed decrease in expression implies that the gap created by irradiation have reached the cell types that express the specific mRNA and an increase indicate reappearance of cell types that express the specific transcript. However, the substantial changes in cellularity must also be taken into consideration, since it severely affects the expression profile when analyzed at the whole tissue level.

From *Dazl *we observed a slight decreased already from day 3 pi. This low level remained until pi day 14, where a steep increase, peaking on pi day 24 was observed. By pi day 31 expression had decreased to pre-treatment level (fig. 3A). As *Dazl *mainly is expressed in B-spermatogonia (from stage VI) and in early spermatocytes [38], the down-regulation at pi day 3 implies that the gap in spermatogenesis had reached the B spermatogonia expressing *Dazl*. However, re-initiation of *Dazl *expression at pi day 14 implies that differentiation of the spermatogonial stem cells (that survived irradiation) had reached the B-spermatogonia stage. This implies that the gap created by irradiation was (14 - 3) 11 days. However, our first measurement was at pi day 3 and the level reached after reconstitution (pi day 31 and on) could indicate that expression at pi day 3 already was reduced. The gap could thus be larger in terms of "spermatogenesis days". The peak expression observed at pi day 17 is, however, most likely further amplified due to changes in the cellularity of the whole testis as elimination of cell types at this point has moved beyond the early spermatocytes and reached the large RNA-rich pachytene spermatocytes. The absence of large RNA-rich pachytene spermatocytes will lead to "artificial" over-representation of all transcripts from other cell types. Likewise, the gradual decrease of *Dazl *from pi day 24 to 31 is likely due to reappearance of the pachytene spermatocytes.

The *Vps26a *transcript is mainly expressed in pachytene (from stage VI) and diplotene spermatocytes but is also present in early round spermatids (Additional file 1). Expression of *Vps26a *after irradiation with 1 Gy decreased from pi day 10 and remained low until pi day 17. At pi day 21, expression however gradually increased (in a bi-modular way) to pre-treatment levels. The observed down regulation at pi day 10 implies that the gap had reached the pachytene spermatocytes that express *Vps26a*. Re-initiation of *Vps26a *expression at pi day 17 again implies that differentiation of spermatogonial stem cells that survived irradiation had reached the mid-pachytene stage. However, if the gap is around 11 days, as suggested by the *Dazl *data, the gap is probably never "big" enough to cover all the *Vps26a *expressing cells as 11 days onwards from mid-pachytene still includes early round spermatids that express *Vps26a*. This is probably also why we observe a short down-regulation-period in expression together with a bi-modular increase. Thus, when the gap has continued beyond round spermatids, mid-pachytene spermatocytes had already reappeared and caused the expression of *Vps26a *to increase again. Even though extrapolation of the gap size from *Vps26a *data is not as clear as the data from *Dazl*, the *Vps26a *profile nevertheless indicate that the gap ranges from at least 7 days (17-10 days) to maximal 14 days (24-10 days; from decrease to "second" increase). Similar extrapolations can be observed from the *Ribc2 *profile, which however was expressed in slightly "later" cells than *Vps26 *(fig 3B).

Contrary to *Vps26a*, *Tnp2 *showed a rather restricted expression. Expression was initiated in haploid round spermatids (stage VI- VII; step 6-7) but was again down-regulated in elongating spermatids (stages XI-XII; step 11-12) (fig 2C and Additional file 2). This narrow expression-window allows a relatively precise estimation of when spermatids are absent and reappear as the testis reconstitutes after irradiation. In mice testis radiated with 1 Gy *Tnp2 *showed a gradually increase until pi day 17. As described for *Dazl*, this up-regulation was most likely due to changes in cellularity originating from the absence of pachytene spermatocytes. This was confirmed by ISH analysis, which showed that the amount of seminiferous tubules containing *Tnp2 *expressing cells remained unchanged until pi day 17, with no signs of an increased expression (fig. 5). Likewise, the gradual decrease of *Tnp2 *from pi day 17 to 24 was probably due to the reappearance of the pachytene spermatocytes diluting the *Tnp2 *transcript. Around pi day 24, the gap reached the *Tnp2 *expressing round and elongating spermatids, which led to a strong decrease in *Tnp2 *expression. This was confirmed by ISH, which showed that *Tnp2 *expressing cell types in the seminiferous tubules were essentially absent from pi day 24 to 28 (fig. 5 & Additional file 3). On pi day 31 *Tnp2 *increased gradually and on pi day 42 it had reached the initial level. Thus, even though *Tnp2 *had a narrow expression window the expression profile was heavily influenced by changes in the cellularity of the reconstituting testis. The actual decrease in *Tnp2 *expression, and thus implicit missing spermatids, happens concurrently with a heavy dilution originating from reappearing pachytene spermatocytes pi day 21 (see *Vps26a *data). From the ISH we know that *Tnp2 *was not present pi day 24 and 28 but down regulation probably happens some days prior and the gap created by irradiation is thus probably around 10 days (partial *Tnp2 *extinction at 21 and subsequently partial reappearance pi day 31). This fits with the *Dazl *data and with the timing of spermatid appearance during normal pn development of the testis [39].

During pn development the expression level of the Sertoli cell specific gene *Gata1 *increased to reach a peak on pn day 12, followed by an apparent down-regulation (fig 2B). The profile follows the percentage of Sertoli cells in the testis [39,40] and the apparent down-regulation nicely correlated with the appearance of large RNA-rich pachytene spermatocytes, while the level of *Gata1 *in the Sertoli cells probably remained constant. After irradiation with 1 Gy, *Gata1 *appeared to be up-regulated from pi day 14 to 24, which coincide with the absence of pachytene spermatocytes and early spermatids. This suggested that the apparent up-regulation likely was caused by changes in cellularity and that the level of *Gata1 *in Sertoli cells probably was not affected by the irradiation but remained constant. In terms of gap size the up-regulation pi day 14 to 24 again indicate a gap size of 10 days.

Using 2 × 1 Gy with a 7 days interval virtually extended the gap with 7 days (fig. 4).

Comparison of the pi profiles to pn profiles indicate that the re-population observed after irradiation was quiet similar to pn induction of spermatogenesis. It takes approximately the same time to generate subsequent cell types pi and pn. In example *Dazl*-expressing cell type-creation takes pn (16 - 4 days) 12 days and pi (24 - 14 days) 10 days (fig. 2A and 3A). *Tnp2 *expressing cell-types development takes pn (40 - 24 days) 16 days and pi (42 - 24 days) 14 days (fig. 2C and 3C). With the inaccuracy from sampling intervals this indicates quiet similar differentiation time with a slight tendency of re-population being faster than pn induction.

In conclusion, the gap created by irradiation with 1 Gy lasted around 10 days and the timing of the reconstituting seminiferous epithelia was similar to that observed during pn induction of spermatogenesis. Some inaccuracy must however be taken into account from sampling intervals and inaccuracy in determining the exact days of gap initiation and termination as the expression profiles are greatly influenced by changes in cellularity of the reconstituting testis.

### Comparison of 1 Gy and 2 × 1 Gy

The 1 Gy and the 2 × 1 Gy irradiation in general created rather similar pi expression profiles. 2 × 1 Gy however yielded an extended period of reduced testis weight and a slightly larger weight loss (fig. 1). Depletion of germ cells in addition seemed more complete as comparison of the variation in expression profiles was more pronounced with the 2 × 1 Gy irradiation (fig. 3 and 4). Looking at pi day 7 for 1 Gy (see above) indicates that virtually no cells had started to reappear at this point. This indicates that reconstitution was not initiated effectively enough to give a sufficient amount of spermatogonia capable of inducing spermatogenesis. The second dose thus essentially eliminates the (few) spermatogonia that were proliferating from spermatogonial stem cells and was "just about to" drive spermatogenesis beyond the first dose. As the large pachytene spermatocytes at pi day 7 with 1 Gy still is present, the elimination of the newly formed spermatogonia probably cannot be observed on the gene expression profiles from the 2 × 1 Gy irradiation. In both cases the sensitive cells seemed to be A_1 _to B spermatogonia.

Application of radiation in treatment of testicular cancer in most cases takes advantage of a fractionated irradiation. As shown here this has a more pronounced effect on germ cell depletion and thus probably also more efficient elimination of cancerous and precursor cells.

## Conclusion

**Figure 6:**
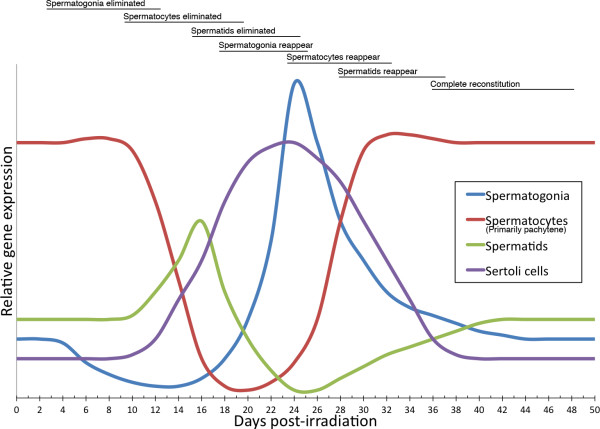
**Schematized gene expression profiles from different cell types**. The curves describes the profile of a gene expressed in the given cell type as observed after irradiation with 1 Gy. Above descriptions of the main cellular events is outlined. The relative intensity of expression signal cannot be compared between cell types and will in addition depend on how abundant the transcripts in question are expressed, while the relative profile should fit if expression is confined to the cell type described. The signal from spermatocytes originates primarily from large pachytene spermatocytes, which has a tremendous effect on the expression signal observed from other cell types.

Irradiation of mice testis created a gap in spermatogenesis, which was initiated by loss of A_1 _to B-spermatogonia. The gap lasted for approximately 10 days and successively extinguished germ cells at different developmental stages. Spermatogonial stem cells were, however, able to re-populate the seminiferous epithelia, which was reconstituted 42 days after irradiation. The gene expression profile of different cell types during reconstitution is schematized in fig. 6. The data show that gene expression can be a useful tool to describe reconstitution of testicular tissue after irradiation or chemotherapy, which otherwise rely on detailed histological descriptions that require carefully trained pathologists.

## List of abbreviations

ISH: *in situ *hybridization; DD: differential display; pi: post-irradiation; pn: post natal.

## Competing interests

The authors declare that they have no competing interests.

## Authors' contributions

FJS carried out DD analysis, interpreted the data and drafted the manuscript. MT carried out all animal work. JEN carried out all ISH analysis. TI, SK, NES, HL and KA all carried out critically revision of important intellectual content and final approval of the version to be published. HL and KA in addition carried out the design, analysis and interpretation of the data and drafted the manuscript. All authors have read and approved the final version of the manuscript.

## Supplementary Material

Additional file 1 Supplementary figure S1: *In situ *hybridization analysis of *Vps26a *- a pachytene spermatocyte-specific gene. Click here for file

Additional file 2 Supplementary figure S2: *In situ *hybridization analysis of *Tnp2 *- a spermatid-specific gene. Click here for file

Additional file 3 Supplementary figure S3: *In situ *hybridization analysis of *Tnp2 *transcript during recovery after irradiation. Click here for file

## References

[B1] Efstathiou E Logothetis C Review of late complications of treatment and late relapse in testicular cancer J Natl Compr Canc Netw 2006 4 10 1059 1070 17112453 10.6004/jnccn.2006.0088

[B2] Russell L Ettlin R Sinha Hikim A Clegg E Histological and histopathological evaluation of the testis 1990 1 Clearwater, FL: Cache River Press

[B3] Meistrich M Effects of chemotherapy and radiotherapy on spermatogenesis Eur Urol 1993 23 1 136 141 8477773

[B4] Shetty G Meistrich M Hormonal approaches to preservation and restoration of male fertility after cancer treatment J Natl Cancer Inst Monogr 2005 34 36 39 10.1093/jncimonographs/lgi002 15784820

[B5] Fosså S Magelssen H Fertility and reproduction after chemotherapy of adult cancer patients: malignant lymphoma and testicular cancer Ann Oncol 2004 15 Suppl 4 iv259 265 15477318 10.1093/annonc/mdh936

[B6] de Rooij D Russell L All you wanted to know about spermatogonia but were afraid to ask J Androl 2000 21 6 776 798 11105904

[B7] Huckins C The spermatogonial stem cell population in adult rats. I. Their morphology, proliferation and maturation Anat Rec 1971 169 3 533 557 10.1002/ar.1091690306 5550532

[B8] Sharpe R Knobil EaN JD Regulation of spermatogenesis The Physiology of Reproduction 1994 2 New York: Raven Press, Inc 1363 1434

[B9] Oakberg E A description of spermiogenesis in the mouse and its use in analysis of the cycle of the seminiferous epithelium and germ cell renewal Am J Anat 1956 99 3 391 413 10.1002/aja.1000990303 13402725

[B10] Oakberg E Gamma-ray sensitivity of spermatogonia of the mouse J Exp Zool 1957 134 2 343 356 10.1002/jez.1401340208 13428958

[B11] Oakberg E Initial depletion and subsequent recovery of spermatogonia of the mouse after 20 r of gamma rays and 100, 300, and 600 r of x-rays Radiat Res 1959 11 700 719 10.2307/3570749 14428127

[B12] Meistrich M Quantitative correlation between testicular stem cell survival, sperm production, and fertility in the mouse after treatment with different cytotoxic agents J Androl 1982 3 58 68

[B13] Meistrich M Wilson G Mathur K Fuller L Rodriguez M McLaughlin P Romaguera J Cabanillas F Ha C Lipshultz L Hagemeister FB Rapid recovery of spermatogenesis after mitoxantrone, vincristine, vinblastine, and prednisone chemotherapy for Hodgkin's disease J Clin Oncol 1997 15 12 3488 3495 9396402 10.1200/JCO.1997.15.12.3488

[B14] Meer Y van der Huiskamp R Davids J Tweel I van der de Rooij D The sensitivity of quiescent and proliferating mouse spermatogonial stem cells to X irradiation Radiat Res 1992 130 3 289 295 10.2307/3578373 1594754

[B15] Meer Y van der Huiskamp R Davids J Tweel I van der de Rooij D The sensitivity to X rays of mouse spermatogonia that are committed to differentiate and of differentiating spermatogonia Radiat Res 1992 130 3 296 302 10.2307/3578374 1594755

[B16] Dym M Clermont Y Role of spermatogonia in the repair of the seminiferous epithelium following x-irradiation of the rat testis Am J Anat 1970 128 3 265 282 10.1002/aja.1001280302 4193812

[B17] Pinon-Lataillade G Maas J Continuous gamma-irradiation of rats: dose-rate effect on loss and recovery of spermatogenesis Strahlentherapie 1985 161 7 421 426 3895586

[B18] Bustos-Obregón E Rodriguez H Testicular x-ray irradiation in adult mice as a model to study spermatogonial proliferation Andrologia 1991 23 6 447 450 1814244 10.1111/j.1439-0272.1991.tb02596.x

[B19] West A Lähdetie J X-irradiation--induced changes in the progression of type B spermatogonia and preleptotene spermatocytes Mol Reprod Dev 2001 58 1 78 87 10.1002/1098-2795(200101)58:1<78::AID-MRD11>3.0.CO;2-J 11144224

[B20] Withers H Hunter N Barkley HJ Reid B Radiation survival and regeneration characteristics of spermatogenic stem cells of mouse testis Radiat Res 1974 57 1 88 103 10.2307/3573759 10874929

[B21] Meistrich M Critical components of testicular function and sensitivity to disruption Biol Reprod 1986 34 1 17 28 10.1095/biolreprod34.1.17 3955133

[B22] van Beek M Meistrich M de Rooij D Probability of self-renewing divisions of spermatogonial stem cells in colonies, formed after fission neutron irradiation Cell Tissue Kinet 1990 23 1 1 16 2302729 10.1111/j.1365-2184.1990.tb01105.x

[B23] Mian T Suzuki N Glenn H Haynie T Meistrich M Radiation damage to mouse testis cells from [99 mTc] pertechnetate J Nucl Med 1977 18 11 1116 1122 915090

[B24] Meistrich M Trostle P Frapart M Erickson R Biosynthesis and localization of lactate dehydrogenase X in pachytene spermatocytes and spermatids of mouse testes Dev Biol 1977 60 2 428 441 10.1016/0012-1606(77)90140-3 924020

[B25] Kreuser E Kurrle E Hetzel W Heymer B Porzsolt F Hautmann R Gaus W Schlipf U Pfeiffer E Heimpel H [Reversible germ cell toxicity following aggressive chemotherapy in patients with testicular tumors: results of a prospective study] Klin Wochenschr 1989 67 7 367 378 10.1007/BF01711264 2501552

[B26] Kangasniemi M Cheng C Toppari J Grima J Stahler M Bardin C Parvinen M Basal and FSH-stimulated steady state levels of SGP-2, alpha 2-macroglobulin, and testibumin in culture media of rat seminiferous tubules at defined stages of the epithelial cycle J Androl 1992 13 3 208 213 1376307

[B27] Rowley M Leach D Warner G Heller C Effect of graded doses of ionizing radiation on the human testis Radiat Res 1974 59 3 665 678 10.2307/3574084 4428015

[B28] Meistrich M Wilson G Brown B da Cunha M Lipshultz L Impact of cyclophosphamide on long-term reduction in sperm count in men treated with combination chemotherapy for Ewing and soft tissue sarcomas Cancer 1992 70 11 2703 2712 10.1002/1097-0142(19921201)70:11<2703::AID-CNCR2820701123>3.0.CO;2-X 1423201

[B29] Zhang Z Shao S Meistrich M The radiation-induced block in spermatogonial differentiation is due to damage to the somatic environment, not the germ cells J Cell Physiol 2007 211 1 149 158 10.1002/jcp.20910 17167785

[B30] Zhang Z Shao S Shetty G Meistrich M Donor Sertoli cells transplanted into irradiated rat testes stimulate partial recovery of endogenous spermatogenesis Reproduction 2009 137 3 497 508 10.1530/REP-08-0120 19036951

[B31] Porter K Shetty G Shuttlesworth G Weng C Huhtaniemi I Pakarinen P Meistrich M Estrogen Enhances Recovery from Radiation-Induced Spermatogonial Arrest in Rat Testes J Androl 2009 in press 19136390 10.2164/jandrol.108.006635

[B32] Jørgensen M Bévort M Kledal T Hansen B Dalgaard M Leffers H Differential display competitive polymerase chain reaction: an optimal tool for assaying gene expression Electrophoresis 1999 20 2 230 240 10197428 10.1002/(SICI)1522-2683(19990201)20:2<230::AID-ELPS230>3.0.CO;2-I

[B33] Nielsen J Hansen M Jørgensen M Tanaka M Almstrup K Skakkebaek N Leffers H Germ cell differentiation-dependent and stage-specific expression of LANCL1 in rodent testis Eur J Histochem 2003 47 3 215 222 14514412 10.4081/830

[B34] Almstrup K Nielsen J Hansen M Tanaka M Skakkebaek N Leffers H Analysis of cell-type-specific gene expression during mouse spermatogenesis Biol Reprod 2004 70 6 1751 1761 10.1095/biolreprod.103.026575 14960480

[B35] Shih D Kleene K A study by in situ hybridization of the stage of appearance and disappearance of the transition protein 2 and the mitochondrial capsule seleno-protein mRNAs during spermatogenesis in the mouse Mol Reprod Dev 1992 33 2 222 227 10.1002/mrd.1080330216 1418993

[B36] Hansen M Nielsen J Tanaka M Almstrup K Skakkebaek N Leffers H Identification and expression profiling of 10 novel spermatid expressed CYPT genes Mol Reprod Dev 2006 73 5 568 579 10.1002/mrd.20463 16477651

[B37] Zhou W Bolden-Tiller O Shetty G Shao S Weng C Pakarinen P Liu Z Stivers D Meistrich M Changes in Gene Expression in Somatic Cells of Rat Testes Resulting from Hormonal Modulation and Radiation-Induced Germ Cell Depletion Biol Reprod 2009 in press 10.1095/biolreprod.109.078048 PMC2802113 19684331

[B38] Huang W Lin Y Hsiao K Eilber K Salido E Yen P Restricted expression of the human DAZ protein in premeiotic germ cells Hum Reprod 2008 23 6 1280 1289 10.1093/humrep/den099 18385127 PMC2902837

[B39] Bellvé A Millette C Bhatnagar Y O'Brien D Dissociation of the mouse testis and characterization of isolated spermatogenic cells J Histochem Cytochem 1977 25 7 480 494 893996 10.1177/25.7.893996

[B40] Ivell R Spiess A Rommerts F, Teerds K Analysing Differential Gene Expression in the Testis Testicular Tangrams 2002 Workshop Supplement 9 Heidelberg: Springer 99 120

