# Supplementary material for: Gene expression profiles of mouse spermatogenesis during recovery from irradiation
Source: Reprod Biol Endocrinol. 2009 Nov 19;7:130. doi: 10.1186/1477-7827-7-130 (PMC2784772; doi:10.1186/1477-7827-7-130)

Supplementary figure S1:

In situ hybridization analysis of Vps26a - a pachytene spermatocyte-specific gene.

Eight representative stages of the seminiferous epithelium are shown. The Vps26a expressing cell types in the different stages are shown schematically at the left side (expressing cells are indicated by red). Illustrations of cell types adapted from Russell et al. [2]. Abbreviations: In, intermediate type spermatogonium; B, type B spermatogonium; PL, pre- leptotene spermatocyte; L, leptotene spermatocyte; Z, zygotene spermatocyte; P, pachytene spermatocyte; D, diplotene spermatocyte; m2Om, meiotic division; 1-16, the steps in the development of the spermatids.

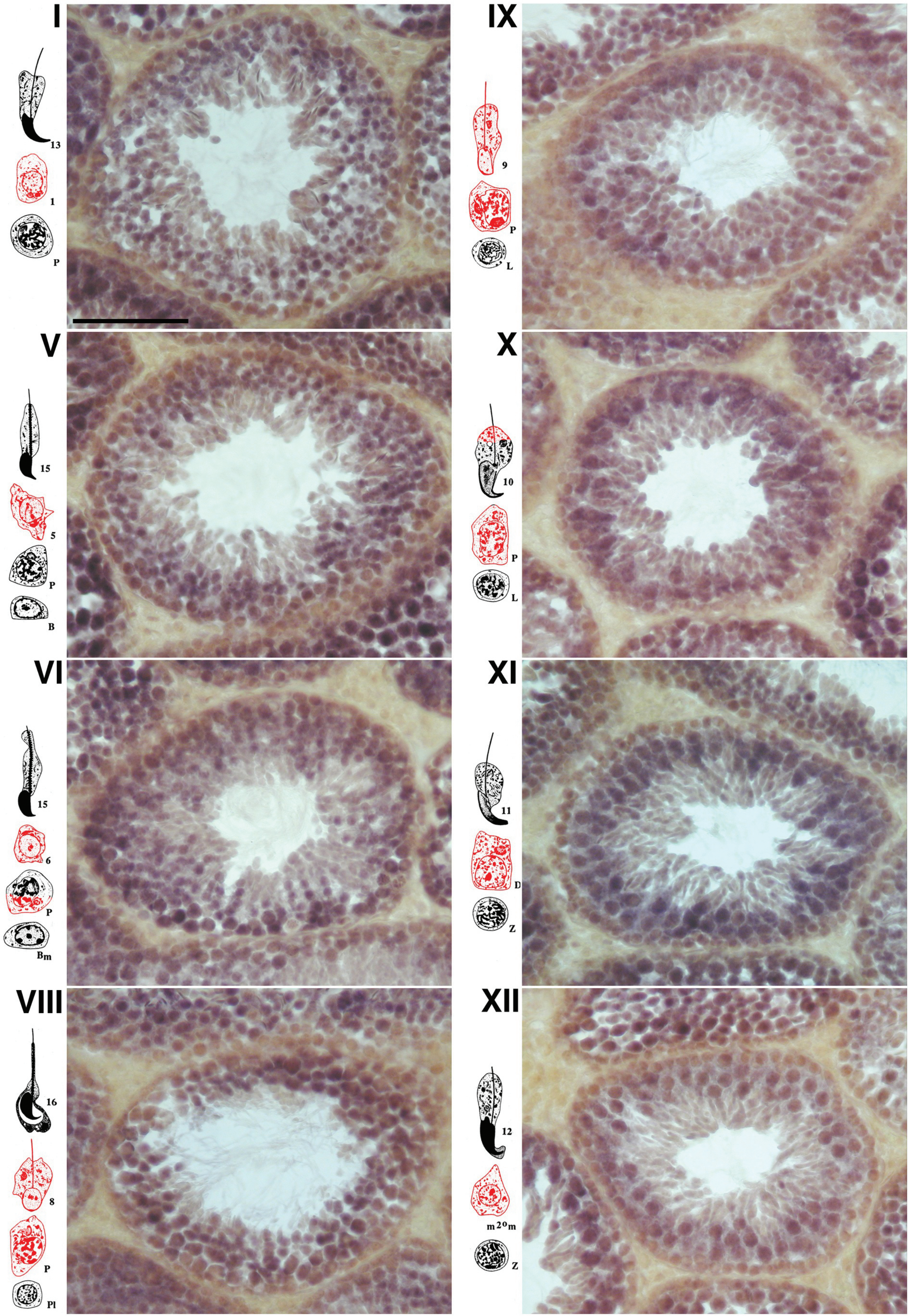

Supplement: Additional file 1 — Supplementary figure S1: In situ hybridization analysis of Vps26a - a pachytene spermatocyte-specific gene. [file 1477-7827-7-130-S1.PDF]
