# Supplementary material for: Gene expression profiles of mouse spermatogenesis during recovery from irradiation
Source: Reprod Biol Endocrinol. 2009 Nov 19;7:130. doi: 10.1186/1477-7827-7-130 (PMC2784772; doi:10.1186/1477-7827-7-130)

Supplementary figure S2:

In situ hybridization analysis of Tnp2 - a spermatid-specific gene.

Representative stages of the seminiferous epithelium are shown and the cell types expressing Tnp2 in different stages are identified as illustrated schematically at the left side of the representative picture (expressing cells are indicated by red). Illustrations of cell types adapted from Russell et al. [2]. Abbreviations as in Additional file 1 (figure S1).

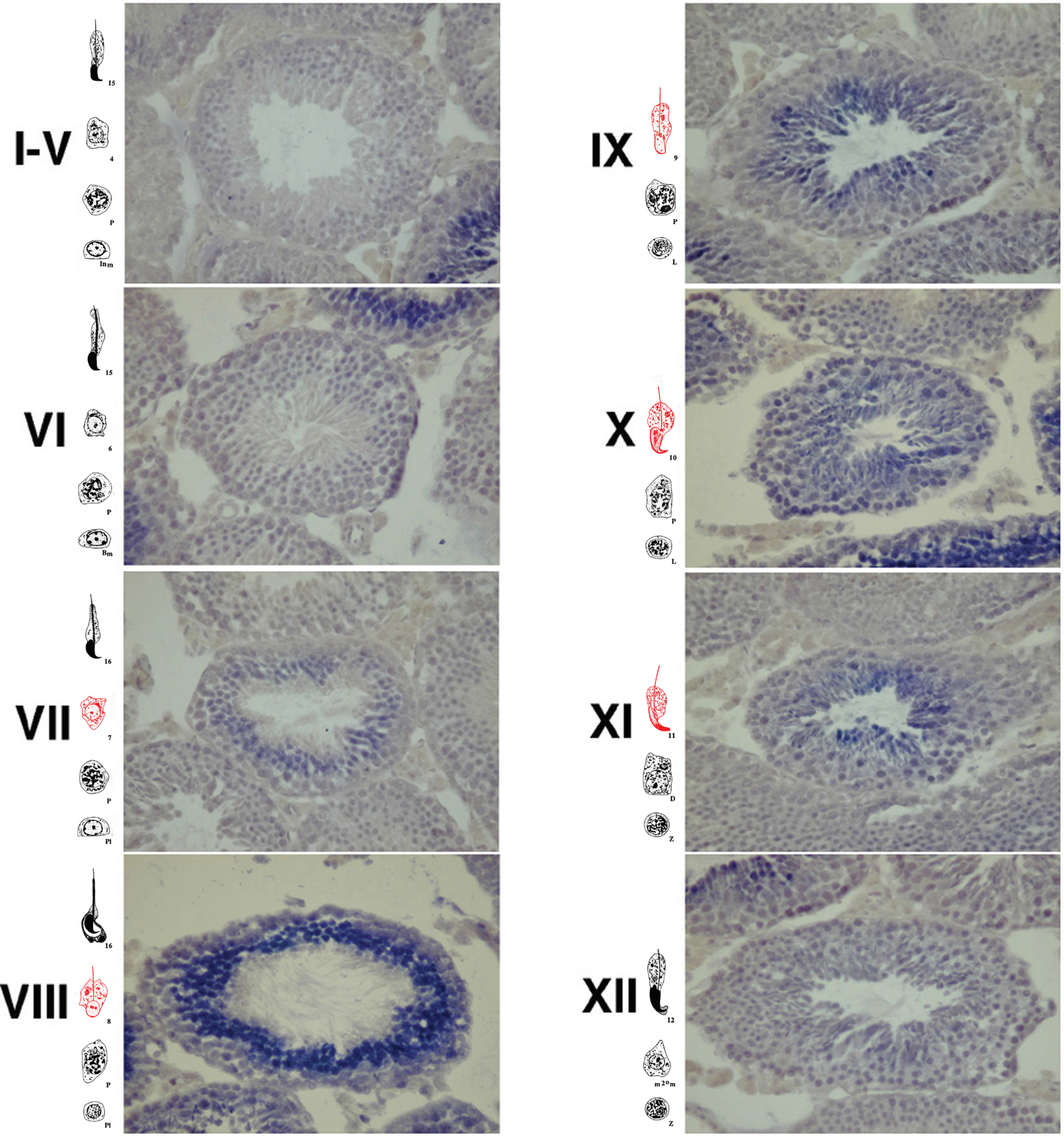

Supplement: Additional file 2 — Supplementary figure S2: In situ hybridization analysis of Tnp2 - a spermatid-specific gene. [file 1477-7827-7-130-S2.PDF]
