# Supplementary material for: Gene expression profiles of mouse spermatogenesis during recovery from irradiation
Source: Reprod Biol Endocrinol. 2009 Nov 19;7:130. doi: 10.1186/1477-7827-7-130 (PMC2784772; doi:10.1186/1477-7827-7-130)

Supplementary figure S3:

In situ hybridization analysis of Tnp2 transcript during recovery after irradiation.

A detailed ISH analysis of Tnp2 in adult mouse testis during the recovery after irradiation with 1 Gy. Low magnification images of whole testis are shown together with a larger magnification of a representative part of the testis. Days after irradiation are indicated on the respective images. Scale bar = 100μm.

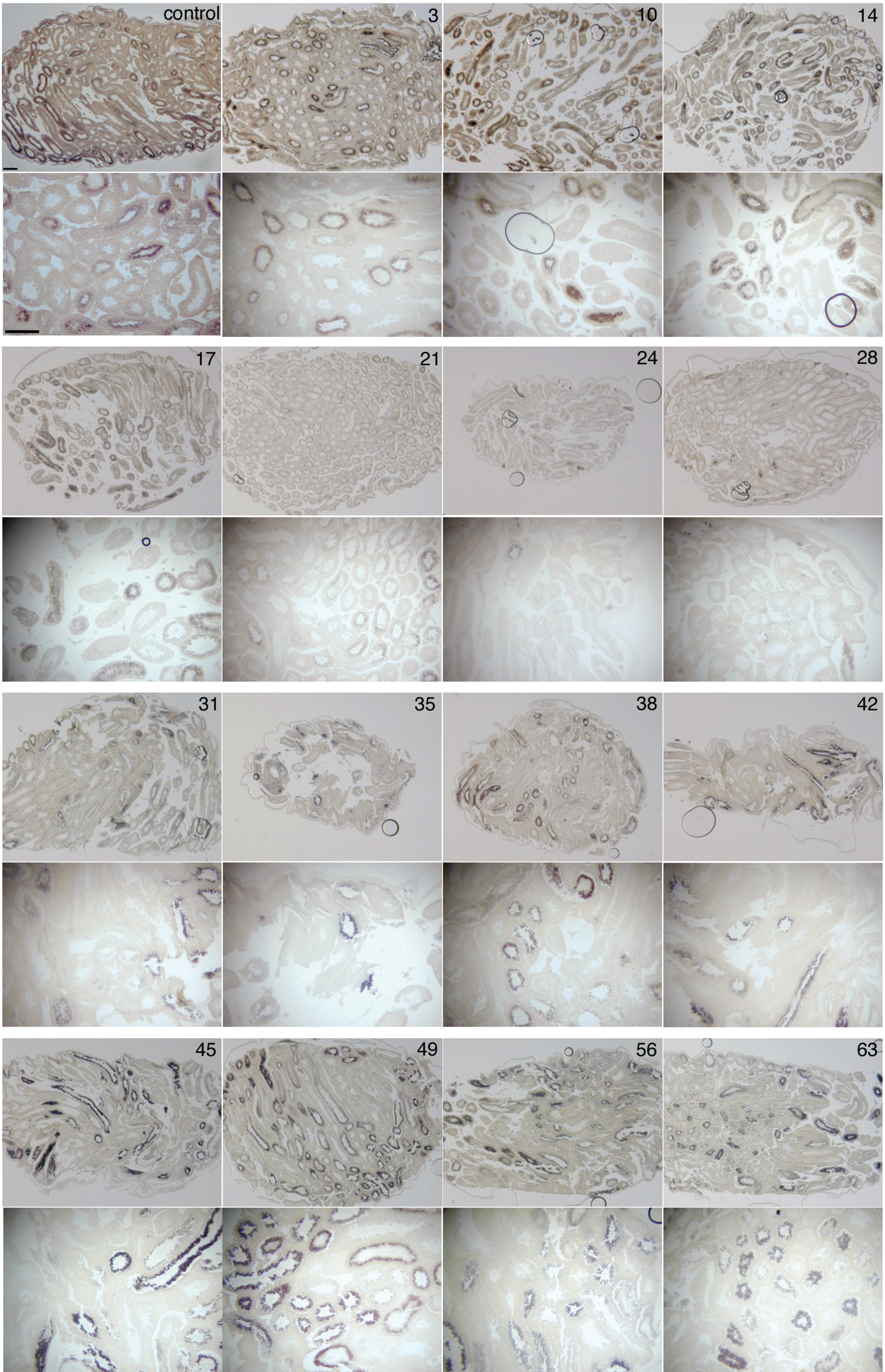

Supplement: Additional file 3 — Supplementary figure S3: In situ hybridization analysis of Tnp2 transcript during recovery after irradiation. [file 1477-7827-7-130-S3.PDF]
